# How Will Work Hour Restrictions Transform the Working Conditions of Resident Physicians?

**DOI:** 10.31662/jmaj.2024-0297

**Published:** 2024-11-25

**Authors:** Kazuya Nagasaki

**Affiliations:** 1 Department of Internal Medicine, Mito Kyodo General Hospital, University of Tsukuba, Tsukuba, Japan

**Keywords:** duty hour restriction, mobile application, time-motion analysis

The issue of long working hours among resident physicians is a global concern, prompting many countries to implement regulations ^(1)^. The main reasons for enforcing work hour restrictions are to ensure patient safety and protect workers’ health and rights, although these justifications vary by country. In Japan, regulations on long working hours were introduced in 2018 to promote worker health and diverse working styles. Physicians and residents were initially exempt from these regulations until April 2024, when they were finally implemented. Under the new rules, all physicians (include most resident physicians) are now limited to a 60-hour workweek, with some exceptions for those in high-intensity training or emergency medicine, who are allowed up to 80 hours per week.

Although these work hours are expected to benefit workers, several concerns remain. A key issue here is the potential impact on resident education. Reducing work hours may limit training opportunities and the number of clinical cases, potentially hindering adequate training. This concern was raised in the early 2000s after work hour restrictions were implemented in the United States and the European Union (EU). In particular, surgical residents experienced a reduction in their clinical experience compared with that before the restrictions. Although the EU’s 48-hour limit had a significant impact, the effect in the U.S. was minimal, with only a small reduction or no change. Among internal medicine residents, there have been reports of reduced time dedicated to educational activities and patient care, although the number of patient encounters and procedural experiences remained unaffected ^(2), (3)^.

A study by Muroya and colleagues, published in the *JMA Journal*, used a mobile application to analyze how residents in three teaching hospitals in Japan spent their time during working hours ^(4)^. This study builds on a 2012 Time-Motion analysis by Deshpande et al., which involved research assistants observing and analyzing residents’ activities ^(5)^. Although this method is accurate, it is costly and labor-intensive to implement regularly. The use of a mobile application allows for less burdensome evaluation. Moreover, the study concluded that compared with data from 10 years ago, the time residents spend on patient care has not decreased and may have even increased. However, the limited time devoted to educational and academic activities remains a concern.

Several factors must be considered when interpreting this study. First, the accuracy of self-assessment using the mobile application was not evaluated. It is crucial to validate this evaluation method by comparing its results with those of other standardized methods. Second, the burden that self-assessment imposes on evaluators (i.e., resident physicians) was not assessed. This issue must be addressed for wider adoption of this method. Additionally, it is premature to conclude that residents are spending more time on patient care over the past decade because the results only reflect the performance of highly motivated teaching hospitals. In Japan, where work hour restrictions are stricter than in the U.S. (60 hours per week vs. 80 hours per week), there is concern that the time spent on patient care could decline. A broader evaluation of residents is necessary, and a validated application would greatly facilitate such assessments.

The impact of work hour restrictions on education is likely to become a major concern for medical professional community in Japan. It is clear that if work hours are limited, the amount of training and other activities will decrease. A future in which patient care time will not decline while educational and academic activities increase is unrealistic. A key factor in medical education is ensuring that the residents we train today can perform well in the future. Metrics like patient volume and time spent on patient care are merely surrogate markers. The critical questions to consider are how work hour restrictions will specifically affect resident training and whether future performance will change as a result. These two evaluations should be conducted together, as one evaluation alone is insufficient. This study will likely play an important role in accurately assessing one of these crucial aspects: changes in training.

## Article Information

### Conflicts of Interest

None

### Author Contributions

All authors have participated in the preparation of the manuscript.
